# Creatine kinase and prognosis in amyotrophic lateral sclerosis: a literature review and multi-centre cohort analysis

**DOI:** 10.1007/s00415-022-11195-8

**Published:** 2022-05-25

**Authors:** Jiali Gao, Thanuja Dharmadasa, Andrea Malaspina, Pamela J. Shaw, Kevin Talbot, Martin R. Turner, Alexander G. Thompson

**Affiliations:** 1 Nuffield Department of Clinical Neurosciences, University of Oxford, John Radcliffe Hospital, West Wing level 3 / level 6, Oxford, OX3 9DU UK; 2 Institute of Neurology, University of London, London, UK; 3 Sheffield Institute for Translational Neuroscience, University of Sheffield, Sheffield, UK

**Keywords:** Motor neuron disease, Creatine kinase, Biomarker

## Abstract

**Background:**

Amyotrophic lateral sclerosis (ALS) is a prognostically heterogeneous neurodegenerative disease. Blood creatine kinase (CK) level has been inconsistently reported as a prognostic biomarker and raised levels in some ALS patients have been presumed to reflect muscle wasting, which is also variable.

**Methods:**

MEDLINE was systematically searched for papers related to CK in ALS and the relevant studies were reviewed. Using data from 222 ALS patients in a multi-centre, prospective, longitudinal cohort, survival analyses using Kaplan–Meier and Cox proportional hazards models were undertaken in relation to CK and other prognostic factors.

**Results:**

Twenty-five studies investigating CK in ALS were identified, of which 10 specifically studied the link between CK and survival. Five studies observed no association, four found that higher CK levels were associated with longer survival and one, the opposite. In our cohort (*n* = 222), 39% of patients had a CK level above the laboratory reference range. Levels were higher in males compared to females (*p* < 0.001), in patients with limb versus bulbar onset of symptoms (*p* < 0.001) and in patients with higher lower motor neuron burden (*p* < 0.001). There was no significant trend in longitudinal CK values. Although a higher standardised log (CK) at first visit was associated with longer survival in univariate analysis (hazard ratio 0.75, *p* = 0.003), there was no significant association after adjusting for other prognostic covariates.

**Conclusion:**

While raised CK levels in ALS do reflect lower motor neuron denervation to a large extent, they are not independently associated with survival when measured in the symptomatic phase of the disease.

**Supplementary Information:**

The online version contains supplementary material available at 10.1007/s00415-022-11195-8.

## Introduction

Amyotrophic lateral sclerosis (ALS) is a fatal neurodegenerative disease characterised by progressive muscle weakness due to degeneration of the upper and lower motor neurons, sharing clinicopathology with frontotemporal dementia. The prognosis of ALS varies greatly between individuals. Although most patients die within 3 years of symptom onset, 10% of patients survive beyond 10 years [1]. Predicting survival by clinical variables alone remains limited at the individual patient level [2]. The search for ALS biomarkers across a range of techniques has yielded several candidates with the potential to improve prognostic stratification [3, 4]. A blood-based biomarker applicable in the routine clinic environment would be valuable.

Creatine kinase (CK) catalyses the reversible conversion of creatine and adenosine triphosphate (ATP) to phosphocreatine and adenosine diphosphate (ADP), an essential component of ATP recycling and energy metabolism [5]. An elevated level of serum CK is usually considered a marker of damage to CK-rich tissues, such as muscle [6]. CK is recognised to be raised in a proportion of ALS patients [7], with a common explanation being that levels reflect secondary muscle breakdown as a result of denervation. CK would represent an attractive biomarker, given its accessibility and relative low expense. However, studies of the association between blood CK and survival in ALS have yielded conflicting results.

We investigated the association between CK levels and survival in ALS through a review of the published literature and analysis of a large, multi-centre, prospective UK cohort, including longitudinal and other prognostic factor data.

## Materials and methods

### Literature review

**Fig. 1:**
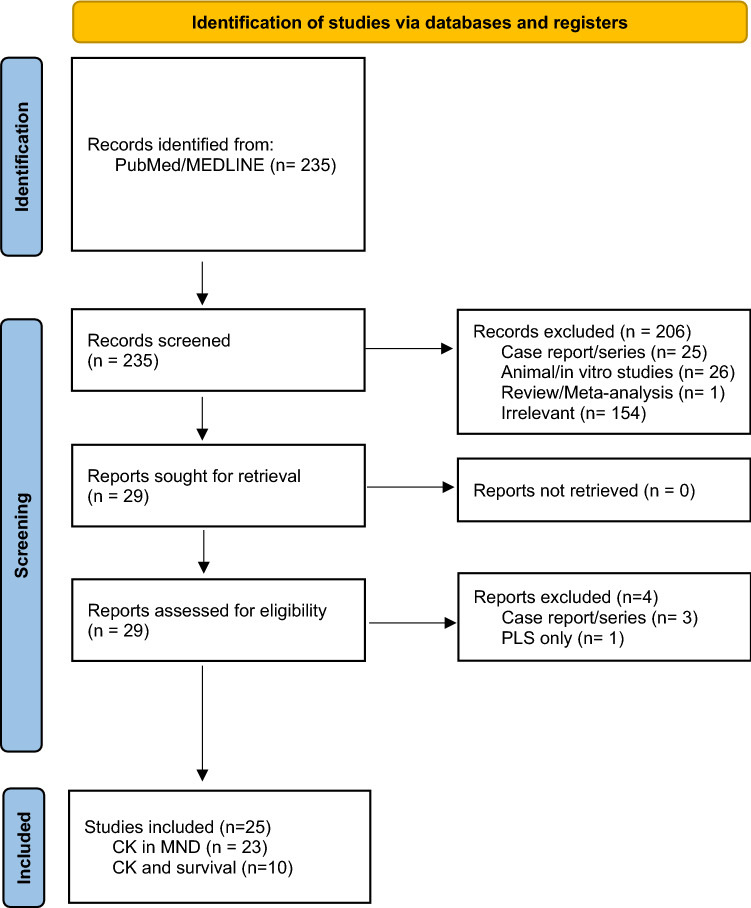
PRISMA flow diagram for study identification

The MEDLINE database was searched on 05/08/21 for articles relating to search terms: “motor neurone disease” or “amyotrophic lateral sclerosis” and “creatine kinase”. The full search strategy can be found in Supplementary Information. Abstracts were screened (JG) and all potentially relevant studies underwent full text review. Foreign language articles, in vitro or non-human studies and reviews, commentaries and editorials reporting non-original data were excluded. Articles reporting original data regarding serum CK levels in ALS patients were included. Figure 1 indicates the flow chart for study selection.

Bibliographic data, study location and timing, sample size, population demographic data, average serum CK levels and the upper laboratory limit for serum CK were extracted from the studies (JG).

Studies including any analyses of CK levels and survival underwent further analyses. From these, the method of survival analysis, the outcome (e.g. hazard ratio or median survival), any confounders included in the model and the overall conclusion were extracted. These articles were quality reviewed and assessed for risk of bias using the Risk of Bias Assessment tool for Non-randomized Studies (RoBANS) by JG [8].

### Patients

‘AMBRoSIA’ (A Multicentre Biomarker Resource Strategy In ALS) is a prospective longitudinal cohort of those attending tertiary ALS referral clinics in Oxford, Sheffield and London, UK. Results of combined biomarker analysis have been published elsewhere [9]. Recruitment commenced in June 2017. Longitudinal data were obtained during routine follow-up clinic visits at intervals of 3–6 months, with a censorship date for survival analyses of 01/02/2020. Survival time was defined as time from symptom onset to death or censorship. Patients with a diagnosis of primary lateral sclerosis were excluded from this analysis.

Venous blood was collected using the BD Vacutainer Safety-Lock set. Serum CK was measured by Clinical Biochemistry laboratories at Oxford University Hospitals NHS Foundation Trust, Sheffield Teaching Hospitals NHS Foundation Trust and Barts Health NHS Trust, London (further details of laboratory assays and normal ranges can be found in Supplementary Information).

Clinical measures were obtained on the same day as biofluid sampling. Symptom onset was defined as first reported muscle weakness. Physical disability was assessed using the revised amyotrophic lateral sclerosis functional rating scale (ALSFRS-R) and forced vital capacity (FVC). Disease progression rate (DPR, points/month), a measure of the slope of decline in ALSFRS-R score, was calculated using the formula [10]:

$$\begin{aligned}&\text{DPR} = (48 - \text{ALSFRS-R score at first visit})/ (\text{months from symptom onset at sampling})\end{aligned}$$

Lean body mass (LBM) was estimated using the Boer formula [11].

To provide a measure of upper versus lower motor neuron signs, clinical lower motor neuron (LMN 0–10) and upper motor neuron (UMN 0–5) burden scores were retrospectively calculated based on the presence of LMN signs (wasting or fasciculation) and UMN signs (hyperreflexia) in five body regions: bulbar, left and right upper, and left and right lower limbs, with one point awarded for the presence of each symptom in each body region. A composite LMN–UMN burden score (–10 to 10) was derived using the equation:

$$\text{LMN UMN burden score} = \text{LMN score}-\left(2 \times \text{UMN score}\right).$$

ALS patient deaths were noted as part of routine healthcare record updating.

### Statistical analysis

Statistical analysis was performed using R version 3.6.1.

Serum CK was not normally distributed. The relationship between baseline CK and other clinical factors was therefore assessed by Spearman rank correlations and Mann–Whitney *U* tests. Spearman correlation coefficients (rho) are presented and are a measure of the strength of the monotonic relationship between two variables on a scale from − 1 to 1.

The common logarithm of CK, log (CK), was judged to be approximately normally distributed by visual inspection of a *Q*–*Q* plot and scaled to a standard normal distribution with a mean of 0 and variance of 1.

Multivariate linear regression models were used to assess for the independent effects of various clinical variables on log (CK). Regression coefficients presented in the results represent the change in standardised log (CK) for a 1-unit increase in that variable, whilst holding all other variables in the model constant.

Longitudinal trends in log (CK) were assessed using linear mixed effect models. The fixed effects of time and of interaction variables on log (CK) were estimated using the ‘lme4’ package for R, with the patient ID modelled as a random intercept. Time was measured both from first sampling and from symptom onset. When measured from symptom onset, data more than 48 months after symptom onset were excluded, due to the paucity of data after this time point.

Survival curves for patients with baseline CK above and below the median were generated using the Kaplan–Meier method and compared using the log-rank test. The relationship between baseline log (CK) values and survival was then analysed using the Cox proportional hazards model and longitudinal values of log (CK) using a time-dependent Cox regression. Multivariate Cox analyses were used to examine the effect of log (CK) on survival adjusting for potential confounders. These included variables associated with CK levels and factors previously associated with survival in ALS for which sufficient data were available: site of initial symptom onset, age, DPR, forced vital capacity, sex and estimated lean body mass (LBM). Survival times were calculated as time from symptom onset to death or census.

Missing data were imputed using multiple imputation by chained equations. Multiple imputed data sets (*n* = 100) were created using predictive mean matching for the continuous variables, logistic regression for two-level factors and a multinomial logit model for multilevel factors, using the ‘mice’ R package [12]. Discarding the first 50 iterations resulted in adequate convergence of all variables. Nelson–Aalen estimators were included in the imputation model [13] and results were pooled using Rubin’s rules [14].

## Results

### Literature review

235 studies were initially identified. Of these, 25 studies were eligible (Fig. 1): 23 of these reported measurements of CK in ALS patients and 10 specifically explored the link between CK and survival (2 articles studied CK and survival without directly reporting CK measurements).

In these studies, the percentage of ALS patients with elevated serum CK ranged from 23 to 90% and mean CK ranged from 63 to 312% of the upper laboratory limit (Supplementary Table 1). With one exception [15], all studies found CK to be higher in male versus female patients and in limb versus bulbar onset, where these variables were investigated.

The longitudinal trend of CK levels in ALS was highly variable. Several studies found no significant trend in values over their respective study periods [15, 16]. Others found an overall decrease in CK [17–19]. One study found that serum CK was elevated prior to the clinical onset of disease in a small sample of eight patients [19]. Various articles also linked higher CK levels with myopathic changes and denervation atrophy on muscle histology [7], increased cramps [16] and increased spontaneous potentials on EMG [20, 21].

In addition, some studies have reported a positive correlation between CK and ALSFRS-R score [19, 22] or that higher CK was associated with slowed progression of the disease [22, 23], but a retrospective study of over 500 patients found no significant correlation [18].

Of the ten studies that specifically probed CK levels in relation to survival in ALS patients, four identified associations of higher CK and longer survival, one an association between lower CK and longer survival and five studies found no association (Table 1). Several studies made no attempt to address potentially confounding covariates (Supplementary Table 2). Baseline characteristics of participants were inconsistently reported, but study populations also varied greatly in terms of average age (from 52.5 to 66.8), average disease duration at study entry (11–27 months), geographical location and follow-up time.

**Table 1:** Studies investigating a link between CK and survival in ALS patients

Study	*n*	Sample	Country	Time period	Analyses	Findings	Conclusion regarding CK and survival
Sinaki et al. [28]	30	Consecutive patients at Mayo Clinic, Rochester	USA	Not reported	1) KM/LR	1) 3-year survival from the date of the examination was 40% for patients with normal CK levels and 37% for patients with elevated CK levels (*p* > 0.43)	No significant association
Gibson et al. [16]	80	Patients enrolled in University of Kentucky’s multicentre nutrition study	USA	2005–2007	1) KM/LR 2) Cox regression	1) Median survival was 4.21 years for patients with baseline CK ≤ 200U/L and 3.31 years for patients with baseline CK > 200U/L (*p* = 0.02) 2) HR for baseline CK > 200U/L versus baseline CK ≤ 200U/L = 1.88, *p* = 0.05. Association holds when Cox model is adjusted for age, gender, race, BIS fat-free mass, location of onset and study site	Lower CK was associated with longer survival
Chio et al. [29]	712	Patients on the Pemonte and Valle d'Aosta Register	Italy	2007–2011	1) KM/LR	1) Median survival was 1.6 years for males with CK ≤ 168 IU (the median value) compared to 1.7 years for males with CK > 168 IU (*p* = 0.914) Median survival was 1.6 years for females with CK ≤ 116 IU (the median value) compared to 2.1 years for females with CK > 116 IU (*p* = 0.128)	No significant association
Rafiq et al. [17]	512	Patients participating in TRO19622 investigational medicinal product trial	Europe	2009–2011	1) Cox regression	1) HR for Log CK = 0.45, *p* < 0.001 Association holds when Cox is model adjusted for site of onset, age, FVC, LBM, disease duration and treatment group	Higher CK was associated with longer survival
Wei et al. [30]	553	Patients registered to the West China Hospital of Sichuan University	China	2009–2014	1) Logistic regression	1) OR for CK and survival beyond 3 years = 1.38, *p* = 0.008	Higher CK was associated with longer survival
Ong et al. [31]	6355	Pooled Resource Open Access ALS Clinical Trials (PRO-ACT) Database	Worldwide	1990–2015	1) Machine learning models	1) Decline in weight, ALP, albumin and CK post-baseline able to predict functional decline class (fast/slow decline in ALSFRS-R score) with AUC = 0.82. Baseline total BR, GGT, urine specific gravity and ALSFRS-R climbing stairs item score (but not CK) able to predict survival class (high/low death risk) with AUC = 0.8	No association found
Lu et al. [23]	95	Not specified	UK	2009–2015	1) Cox regression	1) HR for CK = 0.95, *p* = 0.74	No significant association
Tai et al. [20]	185	Patients registered at Peking Union Medical College Hospital	China	2013–2015	1) KM/LR 2) Cox regression	1) Patients with normal CK levels survived less long than patients with elevated CK levels (*p* = 0.03) 2) HR for Log CK = 0.347, *p* = 0.003. Association holds when Cox model is adjusted for age, site of onset, disease duration, serum creatinine, BMI	Higher CK associated with longer survival
Chen et al. [18]	582	Patients at Sichuan University West China Hospital	China	2008–2018	1) Cox regression	1) HR for Log CK = 0.651, *p* = 0.015. Association holds when Cox model is adjusted for age at disease onset, BMI, ALSFRS-R, disease duration	Higher CK associated with longer survival
Guo et al. [32]	346	Patients enrolled serially at the First Affiliated Hospital of Fujian Medical University	China	2014–2019	1) KM/LR 2) Cox regression	1) Survival for male patients with CK ≤ 177 U/L not significantly different from survival for male patients with CK > 177 U/L (*p* = 0.297). Survival for female patients with CK ≤ 174.5 U/L not significantly different from survival for male patients with CK > 174.5 U/L (p = 0.354) 2) HR for log CK for males = 1.062, *p* = 0.808. HR for log CK for females = 1.165, *p* = 0.730	No significant association

*n* sample size, *KM/LR* Kaplan–Meier/log-rank test, *HR* hazard ratio, *BIS* bioelectric impedance spectroscopy, *OR* odds ratio, *ALP* alkaline phosphatase, *BR* bilirubin, *GGT* gamma glutamyltransferase, *ALSFRS-R* amyotrophic lateral sclerosis functional rating scale revised, *BMI* body mass index

### Multi-centre cohort analysis

Of 247 patients in the AMBRoSIA cohort, 222 had baseline CK measurements. The baseline characteristics for these patients are outlined in Supplementary Table 3. 66% of patients were male, with a median age of 64 years (interquartile range (IQR) 15.8 years) and median disease duration of 18 months (IQR 24.5 months) at study entry. 25% of patients had bulbar onset disease.

### Baseline serum CK

The median baseline CK in this cohort was 191.5 U/L (IQR 219.8U/L). 39% of patients had a CK level above the normal range at baseline, with six patients (< 3%) having a CK above 1000 U/L.

CK levels were correlated with LBM (Spearman’s rho = 0.30, *p* < 0.001). Median CK was significantly higher in males (*p* < 0.001), though not after correcting linearly for LBM, and in patients with limb versus bulbar onset of symptoms (*p* < 0.001, Table 2). Multiple linear regression with LBM, sex and onset site indicated that only LBM was independently associated with log (CK) (*p* = 0.003; onset site *p* = 0.054).

**Table 2:** Serum CK levels and CK/LBM by gender and onset site

Category	*n*	Median CK (U/L)	IQR (U/L)	*P* value
Overall	222	191.5	219.8	
Female	76	143.5	152.3	
Male	146	228.0	238.0	0.0002
Bulbar	52	145.0	133.5	
Limb	159	218.0	249.0	0.0007

Category	*n*	Median CK/LBM	IQR	*P* value
Overall	189	3.66	3.66	
Female	59	3.34	3.42	
Male	124	3.97	4.05	0.345
Bulbar	43	2.82	2.56	
Limb	136	4.05	4.01	0.013

LBM = lean body mass, as estimated by the Boer formula, IQR = interquartile range

*P* values were calculated using Mann–Whitney *U* tests. Cognitive onset was excluded from comparison due to the low number of patients

There was a weak association between higher CK levels and lower disease progression rate (DPR) (Spearman’s rho = − 0.15, *p* = 0.039), which remained after correcting for LBM (Spearman’s rho = − 0.16, *p* = 0.033).

Higher CK levels were also associated with higher LMN as opposed to UMN burden, as estimated by the LMN–UMN burden score (Spearman’s rho = 0.32, *p* < 0.001). Multiple linear regression showed that LMN burden score was positively associated with log(CK) (coefficient = 0.073, *p* = 0.004), whereas UMN burden score was negatively associated with log(CK) (coefficient = − 0.060, *p* = 0.001), when the other is held constant.

### Longitudinal trends in CK

There were 91 patients (41%) in the cohort with longitudinal CK measurements. The baseline characteristics for these patients are outlined in Supplementary Table 2. 75% of patients were male (compared to 68% in the main cohort) and 23% of patients had bulbar onset disease (compared to 25% in the main cohort). Median age was also marginally lower at 62 years rather than 64 years. Other baseline characteristics are broadly similar.

**Fig. 2:**
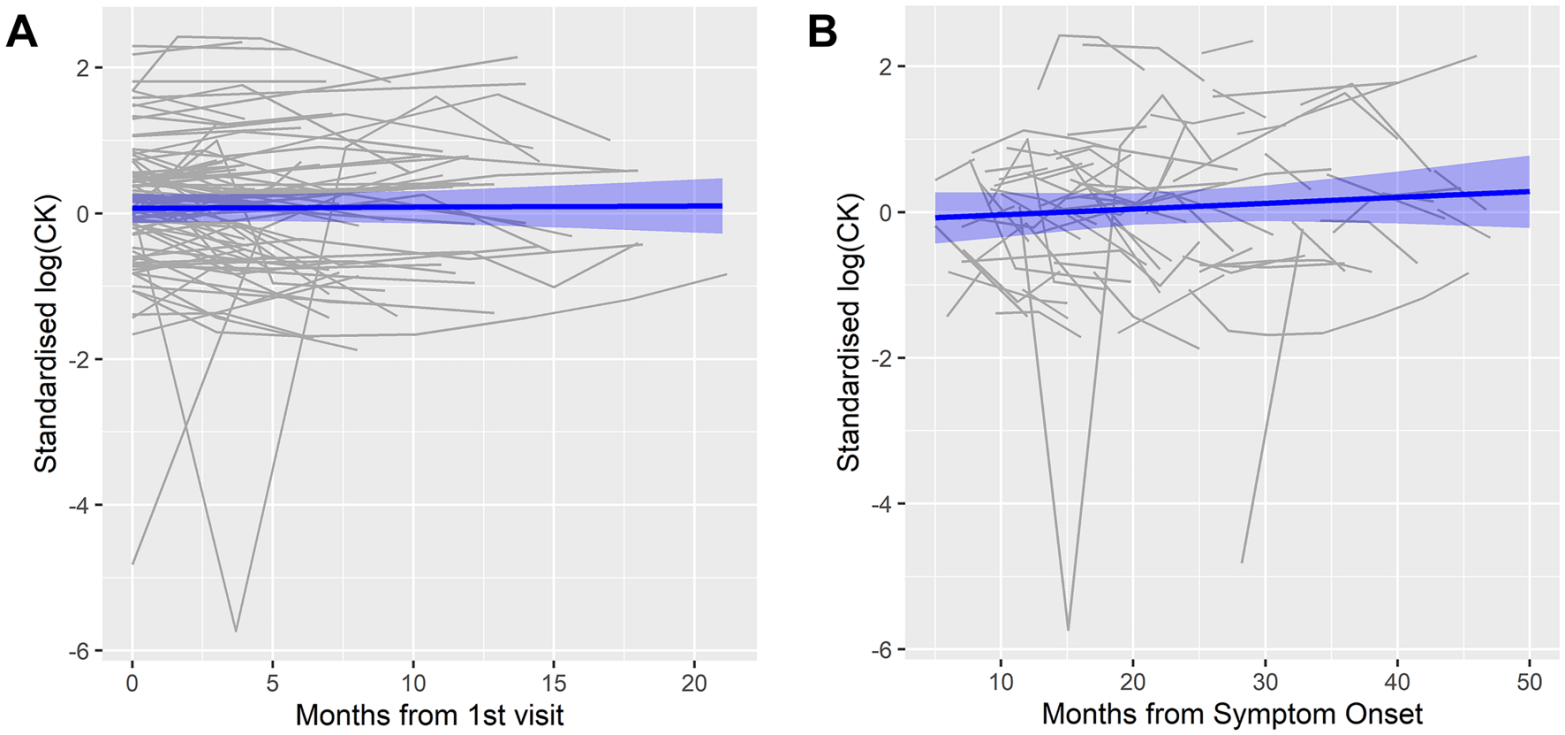
Spaghetti plots of standardised log (CK) values over time in patients who had multiple CK measurements (*n* = 91). **A** Measured from date of first sampling. **B** Measured from symptom onset. The blue lines represent linear mixed effect models fitted to the data and the shaded areas represent the 95% confidence intervals for these models

A linear mixed effects model found no significant temporal trend in standardised log (CK) when measured from date of first sampling (estimate for the effect of time = 0.002, 95% confidence interval − 0.018 ~ 0.021; Fig. 2A) or symptom-onset (estimate for the effect of time = 0.003, 95% confidence interval − 0.003 ~ 0.008; Fig. 2B). As sensitivity analyses, sex, symptom-onset site (limb or bulbar), survival outcome (alive or dead at censorship), the log-transformed disease progression rate (DPR) and the length of survival (survival time above or below the median) were each separately included in the model as interaction variables with time. None of these variables significantly influenced the longitudinal trend of log (CK) (Supplementary Fig. 1).

### CK and survival

**Fig. 3:**
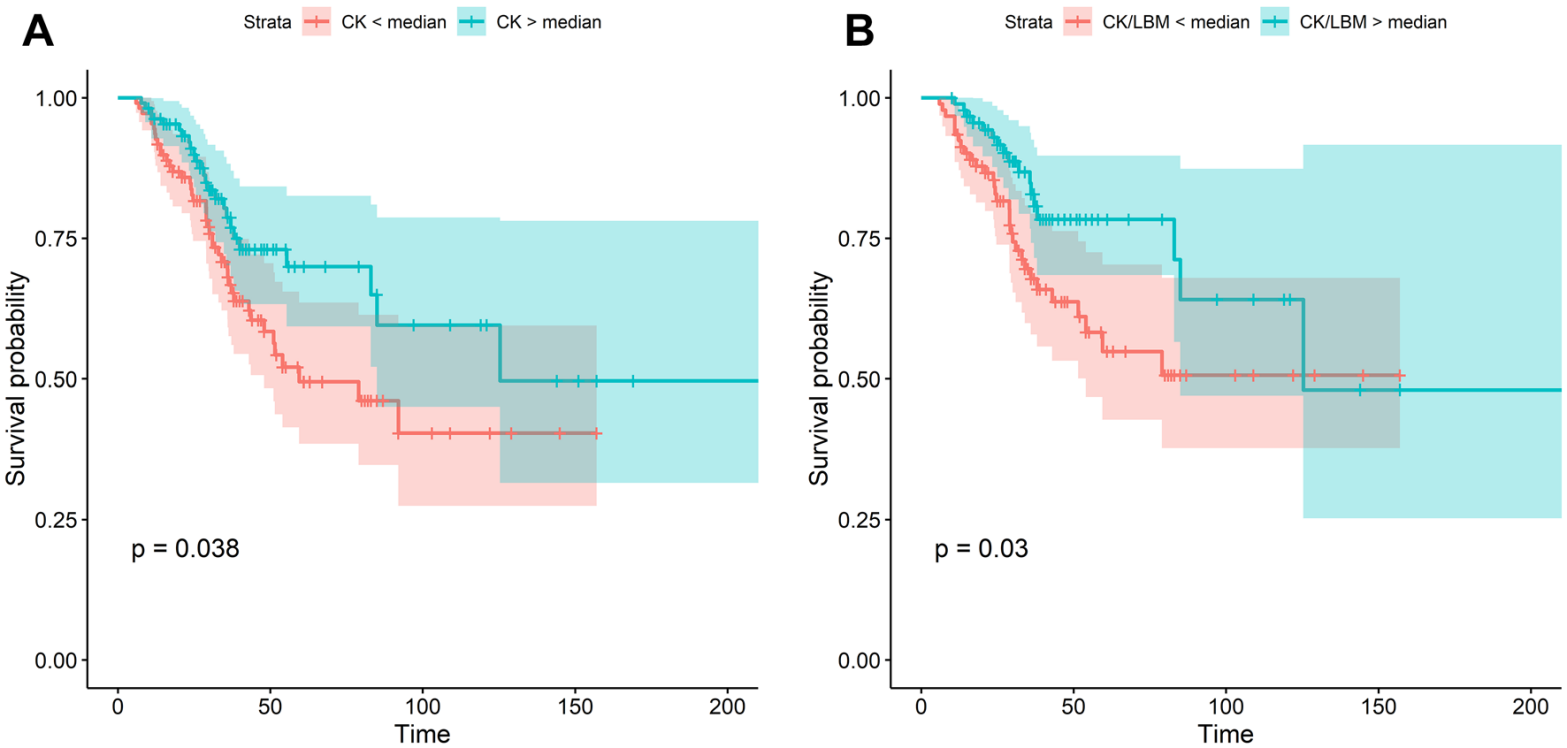
Kaplan–Meier survival curves. **A** For patients with CK levels above and below the median. **B** For patients with CK/LBM above and below the median

Survival data were available for 217 patients (98%) in the cohort with a baseline CK measurement. Kaplan–Meier and log-rank analyses revealed that patients with a baseline CK level higher than the median survived significantly longer than patients with a baseline CK below the median (*p* = 0.038, *n* = 217, 42 events in CK < median group compared to 25 in CK > median; Fig. 3A). When LBM was adjusted for, this effect remained (*p* = 0.030, *n* = 184, 31 events in CK/LBM < median group compared to 17 in CK/LBM > median; Fig. 3B). However, the difference between the survival curves narrowed at later time points when fewer data were available.

Cox proportional hazards regression analysis was also carried out (Table 3). A one standard deviation increase in baseline log (CK) resulted in a 25% reduction in hazard of death. A similar relationship existed for log (CK/LBM). However, the association was not significant when adjusted for other covariates, although fewer patients (*n* = 110) were included in this multivariate analysis due to missing data. To reduce selection bias, multiple imputation was performed. Results were qualitatively similar to complete case analysis with no significant relationship between standardised log (CK) and survival after adjusting for covariates. Inclusion of CK in multivariate models did not markedly improve model fit as measured by the Akaike information criterion (AIC, unimputed 222.1 with CK, 220.3 without; imputed data median AIC with CK 605.9, 605.9 without).

**Table 3:** Cox Proportional hazards analyses using baseline log (CK) and covariates

Model	Variable	HR	*P* value	*n*
1	Standardised log (CK)	0.75	0.003	217
2	Standardised log (CK/LBM)	0.75	0.014	185
3	Standardised log (CK) adjusted	0.90	0.670	108
4	Standardised log (CK) adjusted With imputed data	0.85	0.296	

Models 3 and 4 were adjusted for onset site, age, disease progression rate, forced vital capacity, sex and estimated lean body mass (LBM)

*HR* hazard ratio

To incorporate CK measurements over time, time-dependent Cox regressions were also performed (Table 4). Again, higher log (CK) was not independently associated with significantly longer survival after adjusting for covariates.

**Table 4:** Time-dependent Cox proportional hazards analyses

Model	Variable	HR	*P* value	*n*
1	Standardised log (CK)	0.77	0.010	366
2	Standardised log (CK/LBM)	0.76	0.013	323
3	Standardised log (CK) adjusted	0.69	0.250	146

Model 3 was adjusted for onset site, age, disease progression rate, forced vital capacity, sex and estimated lean body mass (LBM)

*HR* hazard ratio

## Discussion

From a large UK multi-centre, prospective, longitudinal cohort, we conclude that CK values are not independently associated with survival in ALS. Raised levels of CK do seem likely to largely reflect denervation as part of LMN involvement, although the limited sensitivity and potential bias of clinical assessment is acknowledged. Missing data is recognised to limit the power of the multivariate survival analyses to address all potential confounding factors.

From our literature review, previous attempts to characterise the link between CK levels and survival in ALS have produced conflicting results and are limited by the heterogeneity of the study populations.

Our analysis is in line with previous observations that CK is elevated above the laboratory reference range in around two-fifths of ALS patients, with only a very few having levels above 1000U/L. Previously proposed explanations include that raised CK levels reflect secondary muscle breakdown as a result of denervation, thereby indicating the degree of lower motor neuron (LMN) involvement, or that CK may be upregulated in response to metabolic stress as part of a protective mechanism or response to hypermetabolism in ALS [16, 17, 24]. Higher CK levels have also been correlated with muscle cramps [16].

Consistent with previous reports, CK levels increase with increased estimated lean body mass in our cohort [17, 25]. This is assumed to be explained by patients with greater muscle mass having larger pools of CK within their muscles to release. CK levels are higher in male compared to female patients, and in patients with limb rather than bulbar onset of symptoms. Our adjustment for LBM suggests that the difference in CK between male and female patients is likely to be accounted for by differences in muscle mass. This is supported by a previous study in which adjusting for bioelectric impedance spectroscopy measurements negated the difference in CK levels between sexes [16].

In contrast, the difference in CK levels between bulbar and limb-onset patients remained after correcting for LBM. This is assumed to reflect a larger volume of muscle involvement, and thus region of denervation, in limb-onset patients. This theory is supported by our finding that CK levels are associated with greater LMN than UMN clinical burden and by previous reports that CK levels correlate with spontaneous potentials on EMG, a measure of denervation [20, 21].

We found no overall longitudinal trend in CK levels (including in subgroup analysis), in contrast to some studies which have reported a decrease over time [17–19]. Given that CK levels were positively correlated with LBM, a decrease in CK over time might be expected to reflect decreasing muscle mass with disease progression. However, all of these analyses are limited by the clinical heterogeneity of ALS, by which there are patients of variable disease duration and muscle wasting at study entry, and so less power to detect more complex associations with CK levels over time. The one study to measure CK levels prior to disease onset [19] provides some evidence that CK levels may be elevated prior to the clinical onset of symptoms (generally within 12 months prior to symptoms, consistent with the timing of the rise seen in plasma neurofilament and CSF chitinase levels [26, 27]). This suggests that CK levels may retain important value in the pre-symptomatic monitoring of genetically at-risk populations, and this warrants further study. The relative stability of CK in the natural history of ALS progression is also potentially still valuable in the broader concept of pharmacodynamic biomarkers, including interim decision points in therapeutic trials.

## Supplementary Information

Below is the link to the electronic supplementary material.

Supplementary file1 (DOCX 1277 KB)
