# Supplementary material for: Creatine kinase and prognosis in amyotrophic lateral sclerosis: a literature review and multi-centre cohort analysis
Source: J Neurol. 2022 May 25;269(10):5395–404. doi: 10.1007/s00415-022-11195-8 (PMC9467954; doi:10.1007/s00415-022-11195-8)
Supplement: Supplementary file 1 — Supplementary file1 (DOCX 1277 KB) [file 415_2022_11195_MOESM1_ESM.docx]

**Supplemental information**

**Creatine kinase and prognosis in amyotrophic lateral sclerosis: a literature review and multi-centre cohort analysis**

Jiali Gao^1^

Thanuja Dharmadasa^1^

Andrea Malaspina^2^

Pamela J Shaw^3^

Kevin Talbot^1^

Martin R Turner^1*^

Alexander G Thompson^1*^

^1^Nuffield Department of Clinical Neurosciences, University of Oxford, UK

^2^­Institute of Neurology, University of London, UK

^3^Sheffield Institute for Translational Neuroscience, University of Sheffield, UK

*Correspondence:

Dr Alex Thompson Prof Martin Turner

[alexander.thompson@ndcn.ox.ac.uk](about:blank) [martin.turner@ndcn.ox.ac.uk](about:blank)

West Wing Level 3 West Wing Level 6

John Radcliffe Hospital John Radcliffe Hospital

Oxford OX3 9DU Oxford OX3 9DU

UK UK

## MEDLINE search term for literature review

(“creatine kinase*”[tiab] OR CK [tiab] OR “creatine phosphokinase*”[tiab] OR CPK [tiab] or “phosphocreatine kinase*”[tiab] OR creatine kinase[mh])

AND

(“Motor neuron? disease”[tiab] OR MND [tiab] OR Amyotrophic lateral sclerosis [tiab] OR ALS [tiab] OR “Gehrig?? Disease”[tiab] OR “Lou?Gehrig?? Disease”[tiab] OR Motor Neuron disease [mh])

AND

(english[Filter])

## Laboratory assays

In Oxford and Sheffield, plasma CK was measured using the Abbott Architext c16000 general chemistry analyser using an enzymatic N-acetyl-L-cysteine (NAC) method; reproducibility was 1.7% at 81 U/L and 1.4% at 405 U/L. In London, CK was measured using the Beckman Coulter CK-NAC assay on the AU5800 analyser; reproducibility was 1.5% at 117 U/L and 2.5% at 452 U/L.

The laboratory normal range for CK was 29-200 U/L in Oxford and 40-320 U/L in Sheffield and London.

## Supplementary table 1 Studies measuring CK in MND patients

| Study | n | Sample | Country | Time period | Ratio of mean CK/ upper lab limit for CK* | Abnormal CK (%) | Other findings |
| --- | --- | --- | --- | --- | --- | --- | --- |
| Williams ER and Bruford A 1970[1] | 46 | Not specified | UK | 1965-1969 |  | 59 |  |
| Welch KM and Goldberg DM 1972[2] | 21 | Patients admitted to Sheffield Royal Hospital | UK | 1966-1970 |  | 52 | 10/11 pts with abnormal CK were within the first 12 months of the disease |
| Edmonds PJ and Ziegler DK 1975[3] | 10 | Patients at University of Kansas Medical Center | USA | 1967-1972 |  | 90 |  |
| Amrit AN and Anderson MS 1974[4] | 94 | Patients admitted to Methodist Hospital | USA | 1970-1973 |  | 76 | CK was higher in patients with shorter disease duration  CK was higher in patients with myopathic changes and denervation atrophy on muscle histology |
| Harrington TM et al. 1983[5] | 100 | Patients seen at Mayo Clinic, Rochester | USA | None reported | 3.12 | 43 | A higher proportion of patients with elevated CK were spinal onset than those with normal CK. |
| Sinaki M and Mulder DW 1986[6] | 30 | Consecutive patients at Mayo Clinic, Rochester | USA | None reported |  | 50 |  |
| Felice KJ and North WA 1998[7] | 140 | Patients at the University of Connecticut School of Medicine | USA | 1993-1998 | 1.22 | 41 | CK was higher in males verses females CK was higher in spinal onset verses bulbar onset patients  CK was not significantly different in sporadic verses familial ALS  CK correlated poorly with age of onset |
| Lima AF et al. 2003[8] | 87 | Patients at the Centro de Estudos Egas Moniz | Portugal | 1997-2003 | 1.57 | 43 | CK was not significantly different in males verses females  CK was not significantly different in spinal verses bulbar onset CK did not significantly change between first and 2nd measurement (3-9 months later)  CK was not significantly correlated with number of fasciculations clinically or on EMG or the number of muscles with fibrillation |
| Sussmuth SD et al. 2003[9] | 20 | Patients at the University Hospital of Ulm | Germany | None reported |  | 70 | CK was mainly normal in the earliest disease stages, increase then peaks between months 12 and 30 |
| Ilzecka J and Stelmasiak Z 2003[10] | 30 | Not specified | Poland | None reported | 0.63 | 43 | CK was not significantly different between groups split by age, sex, duration of disease or Munsat ALS score CK was higher in spinal verses bulbar onset |
| Gibson SB et al. 2015[11] | 80 | Patients enrolled in University of Kentucky’s multicenter nutrition study | USA | 2005-2007 | 1.11 | 45 | No significant temporal trend in CK over 48 weeks CK was higher in males verses females, but not after adjusting for fat free mass.  CK was higher in limb verses bulbar onset CK was positively correlated with cramp index but not spasticity or fasciculations |
| Chahin N and Sorenson EJ 2009[12] | 36 | Patients at Mayo Clinic, Rochester | USA | 2001-2008 | 0.90 | 23 |  |
| Zhang Y et al. 2011[13] | 119 | Patients at First Affiliated Hospital | China | 2006-2011 | 1.51 |  |  |
| Chio A et al. 2014[14] | 712 | Patients on the Pemonte and Valle d'Aosta Register | Italy | 2007-2011 | 1.14 (male) 0.88 (female) |  |  |
| Rafiq MK et al. 2016[15] | 512 | Patients participating in the TRO19622 investigational medicinal product trial | Europe | 2009-2011 | 1.41 |  | CK was not significantly correlated with disease duration  CK decreased over 18 months by 1.3% per month CK correlated weakly positively with manual muscle scores and strongly positively with LBM and serum creatinine  CK was not correlated with ALSFRS-R limb function domains |
| Lu CH et al. 2016[16] | 95 | Not specified | UK | 2009-2015 | 0.98 (median) |  | CK was positively correlated with IL-5 levels  CK was higher in patients with lower progression rate and earlier disease stage |
| Tai H et al. 2017[17] | 185 | Patients registered at Peking Union Medical College Hospital | China | 2013-2015 | 1.19 | 43 | CK was higher in males verses females CK was higher in spinal verses bulbar onset CK was weakly positively correlated with creatinine, EMG spontaneous potentials score, duration of illness, but not with age or BMI |
| Mirian A and Korngut L 2018[18] | 85 | Patients at University of Calgary, Neuromuscular Program | Canada | 2012-2016 |  | 35 |  |
| Tai H et al. 2018[19] | 238 | Consecutive patients registered at Peking Union Medical College Hospital | China | 2013-2016 |  | 38 | CK was higher in males verses females CK was positively correlated with the mean spontaneous potential score and F wave persistence on EMG but not with the compound muscle action potential sum score. |
| Chen XP et al. 2021[20] | 582 | Patients at Sichuan University West China Hospital | China | 2008-2018 | 0.93 |  | CK was higher in males verses females CK was higher in spinal verses bulbar onset CK was positively correlated with disease duration but not with ALSFRS-R score, BMI or progression rate CK decreased over >6 months |
| Ito D et al. 2019[21] | 81 | Patients at Nagoya University Hospital | Japan | 2013-2018 | 1.22 |  | CK was higher in spinal verses bulbar onset  CK decreased over 48 weeks  LogCK was weakly positively correlated with ALSFRS-R CK became elevated prior to disease onset in 8 subjects with prior values |
| Guo QF et al. 2021[22] | 346 | Patients enrolled serially at the First Affiliated Hospital of Fujian Medical University | China | 2014-2019 | 0.78 (median) |  | CK was higher in males verses females No clear trend in CK over >12 months |
| Ceccanti M et al. 2020[23] | 126 | Patients recruited consecutively at Sapienza University of Roma and San Camillo Forlanini Hospital | Italy | 2017-2019 | 1.38 |  | CK was higher in spinal verses bulbar onset CK was higher in slow progressive patients verses fast progressive patients  CK was logarithmically correlated with ALSFRS-R score |

*Where no upper laboratory limit is quoted in the article, 182.5U/L was used; where separate upper laboratory limits were quoted for men and women, these were averaged.

n= sample size; LBM = lean body mass; ALSFRS-R = Amyotrophic lateral sclerosis functional rating scale revised; BMI = body mass index; CK = creatine kinase

## Supplementary table 2 – risk of bias assessments using the RoBANS tool

| Study | Selection of Participants | Confounding variables | Measurement of exposure | Blinding of outcome assessment | Incomplete outcome data | Selective outcome reporting |
| --- | --- | --- | --- | --- | --- | --- |
| Sinaki M et al. 1986 | Low | High | Low | Low | Uncertain | Uncertain |
| Gibson SB et al. 2015 | Low | Low | Low | Low | Uncertain | Uncertain |
| Chio A et al. 2014 | Low | High | Low | Low | Uncertain | Uncertain |
| Rafiq MK et al. 2016 | Low | Low | Low | Low | Uncertain | Uncertain |
| Wei QQ et al. 2018 | Low | High | Low | Low | Low | Uncertain |
| Ong ML et al. 2017 | Low | Low | Low | Low | Low | Uncertain |
| Lu CH et al. 2016 | Low | Low | Low | Low | Uncertain | Uncertain |
| Tai H et al. 2017 | Low | Low | Low | Low | Low | Uncertain |
| Chen XP et al 2021 | Low | Low | Low | Low | Uncertain | Uncertain |
| Guo QF et al. 2021 | Low | Low | Low | Low | Low | Uncertain |

## Supplementary Table 3 – Baseline characteristics

| Characteristic | Number (% total) *or* Median ± IQR | |
| --- | --- | --- |
|  | **Patients with baseline CK (n=222)** | **Patients with longitudinal data (n=91)** |
| Gender - female | 76 (34%) | 23 (25%) |
| Gender - male | 146 (66%) | 68 (75%) |
| Onset Site - Bulbar | 52 (25%) | 21 (23%) |
| Onset Site - Spinal | 159 (74%) | 69 (77%) |
| Age (years) | 64 ± 15.8 | 62 ± 14.0 |
| Weight (kg) | 74 ± 20.0 | 75 ± 19.1 |
| BMI | 24.7 ± 4.8 | 24.3 ± 3.9 |
| Disease duration at study entry (months) | 18 ± 24.5 | 18.8 ± 23.4 |
| ALSFRS-R | 39 ± 10 | 40 ± 9 |
| Disease progression rate | 0.4 ± 0.6 | 0.4 ± 0.4 |
| ALS-specific ECAS | 86 ± 12 | 86 ± 10 |
| FVC | 84 ± 36.5 | 87 ± 27.0 |
| Riluzole use | 75 (37%) | 38 (42%) |

## Supplementary Figure 1 – Spaghetti plots of standardised log (CK) values over time in patients who had multiple CK measurements (n=91), split by additional variables

The blue lines represent linear mixed effect models fitted to the data and the shaded areas represent the 95% confidence intervals for these models.
Long survivors are defined as individuals with survival times above the median and short survivors as those with survival times equal to or below the median

**A** – measured from date of first sampling


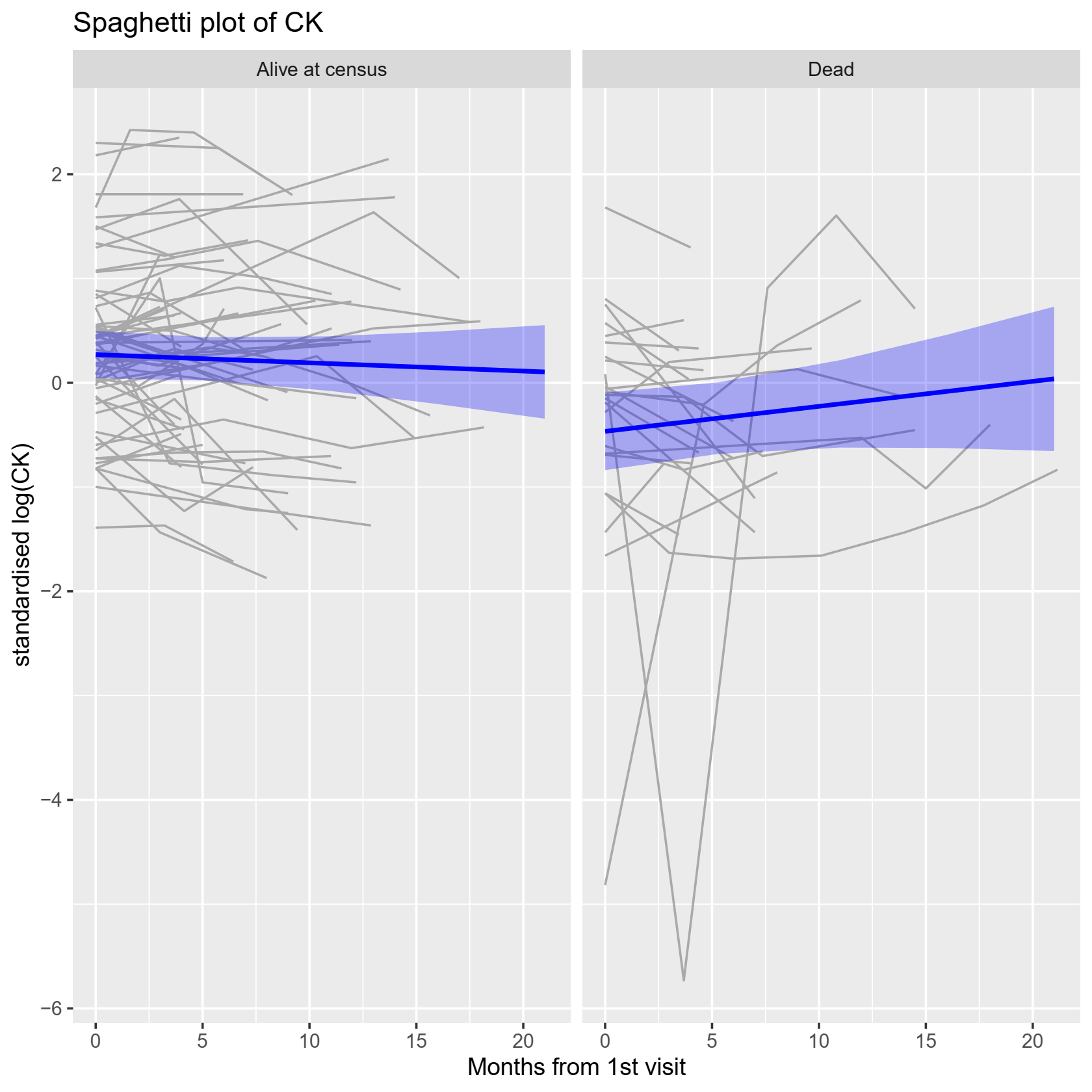

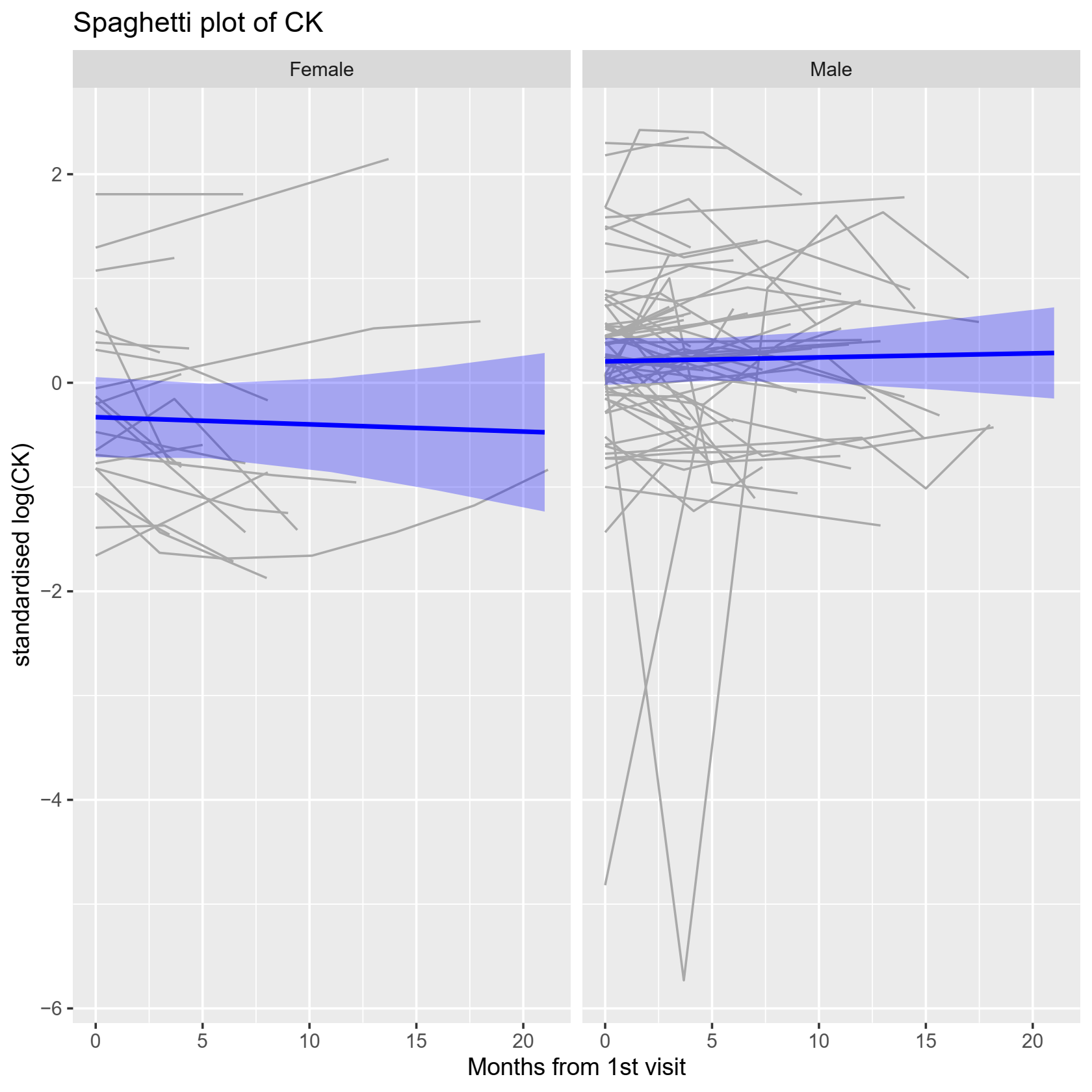


Estimate for fixed effect of Survival status x Time Estimate for fixed effect of Sex x Time
= 0.032 (95% CI -0.011 ~ 0.075) = 0.011 (95% CI -0.035 ~ 0.056)


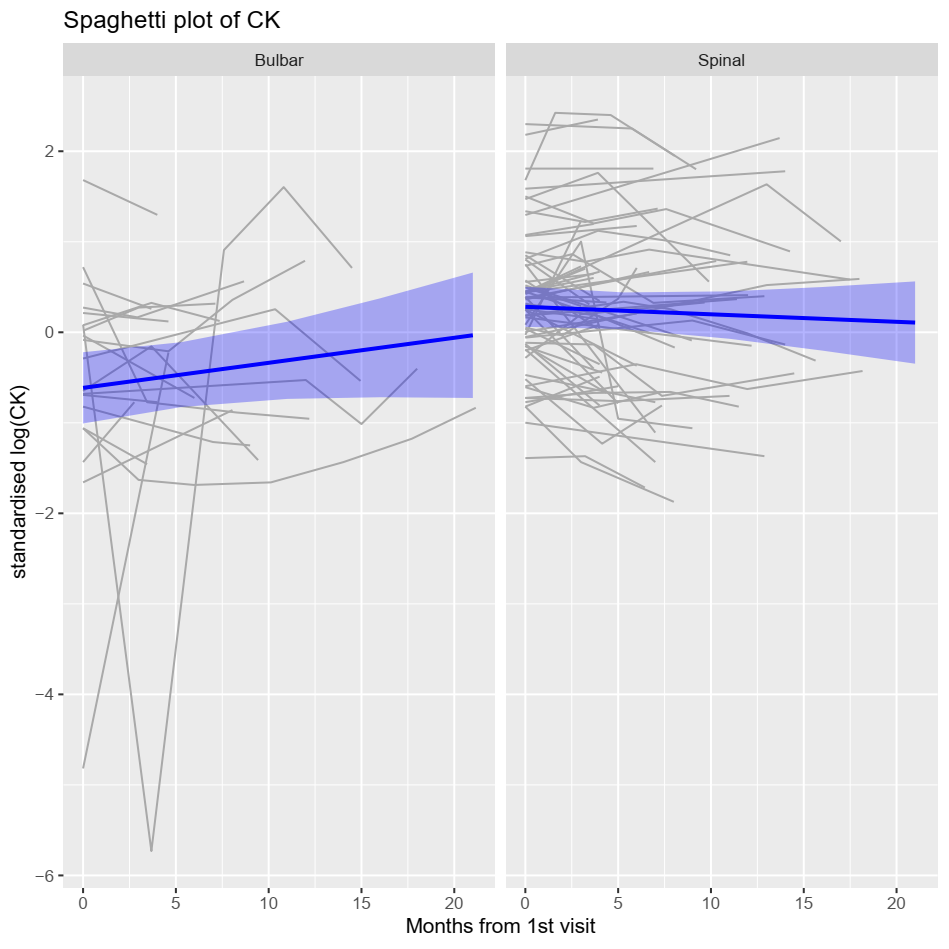

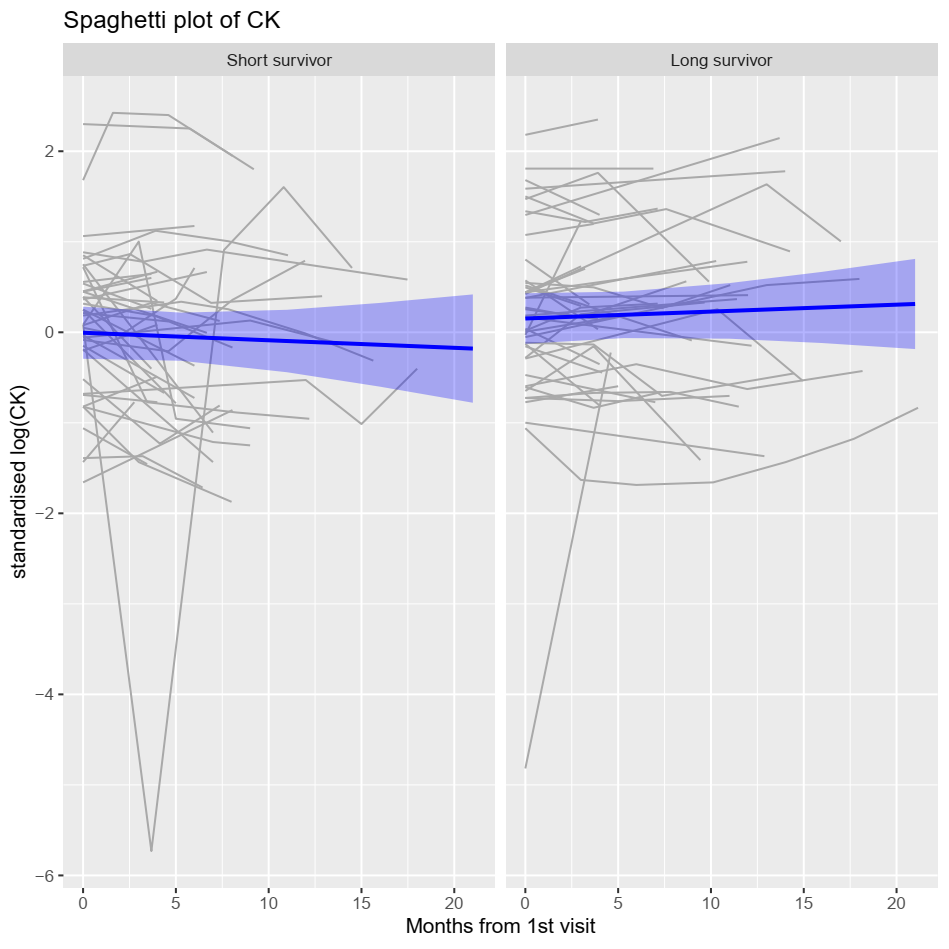


Estimate for fixed effect of Onset site x Time Estimate for fixed effect of Survival length x Time
= -0.036 (95% CI -0.079 ~ 0.007) = 0.016 (95% CI -0.025 ~ 0.056)

**B** – measured from symptom onset (data >48 months excluded)


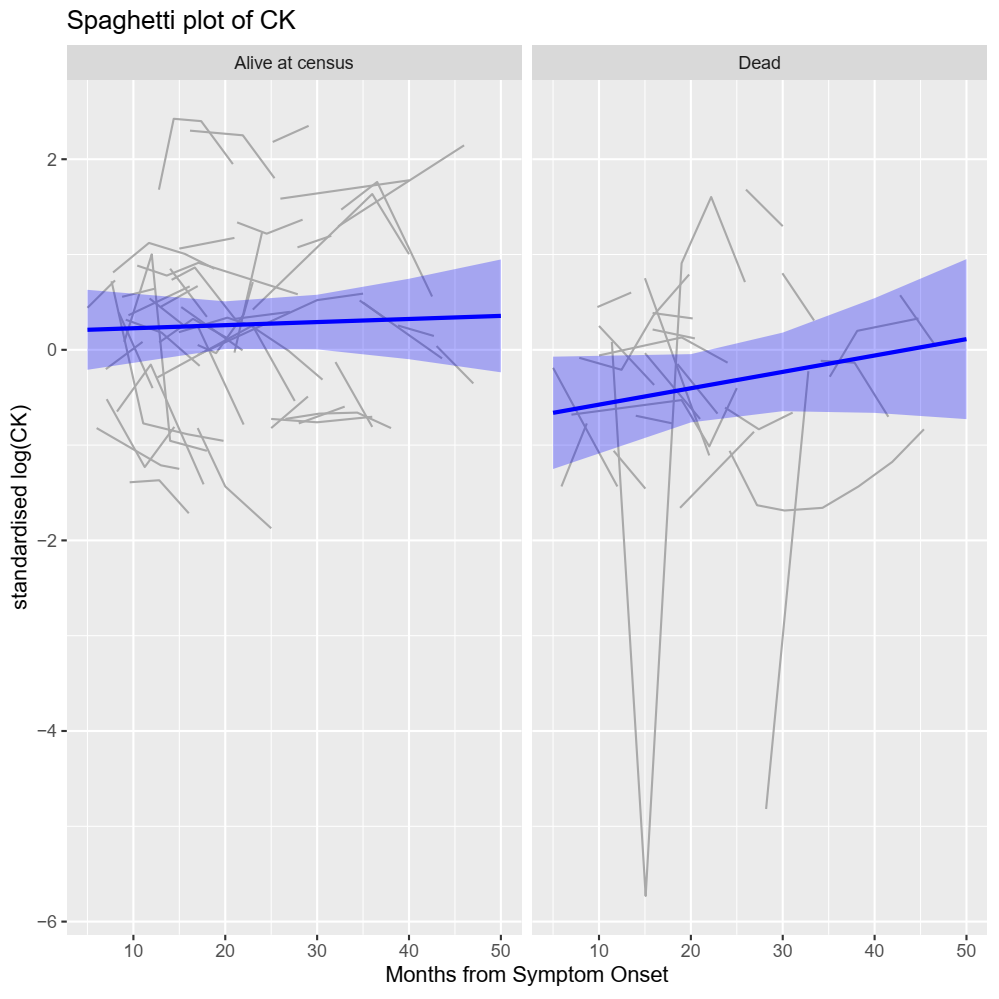

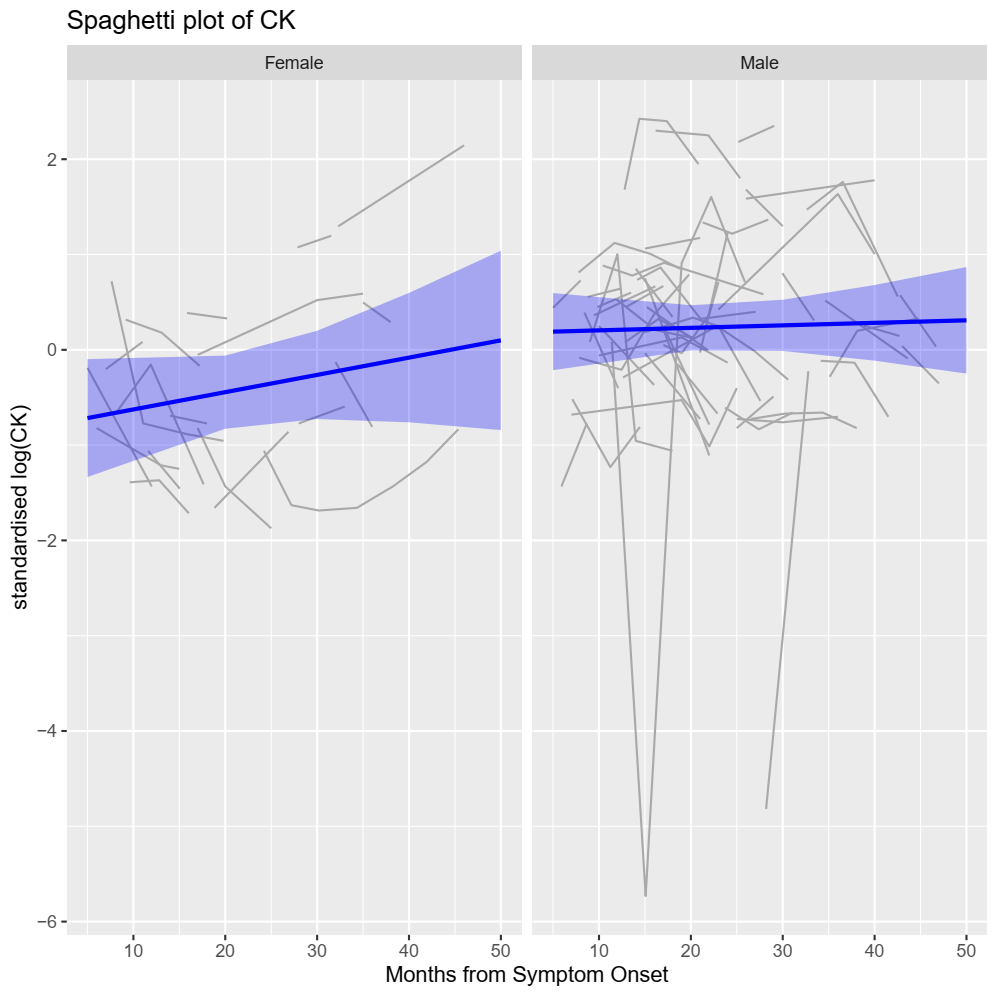


Estimate for fixed effect of Survival status x Time Estimate for fixed effect of Sex x Time
= 0.0314 (95% CI -0.020 ~ 0.048) = -0.015 (95% CI -0.051 ~ 0.020)


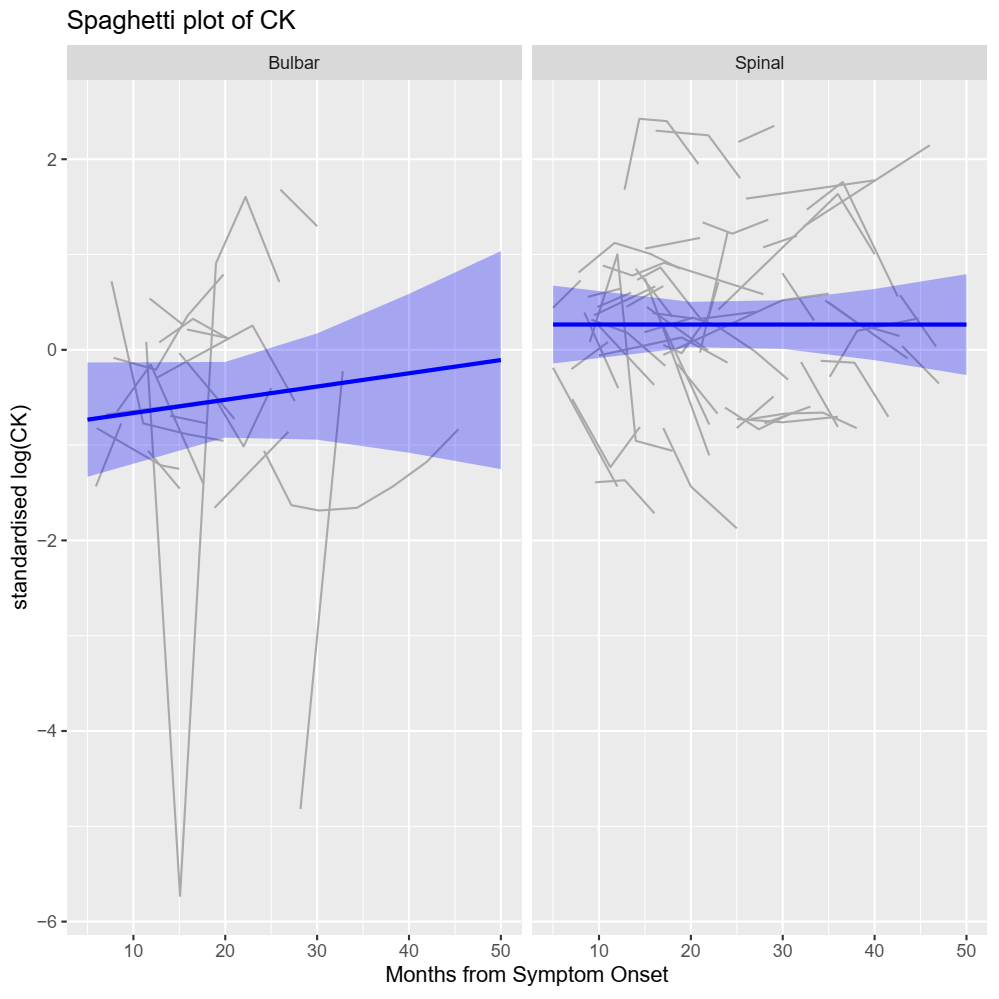

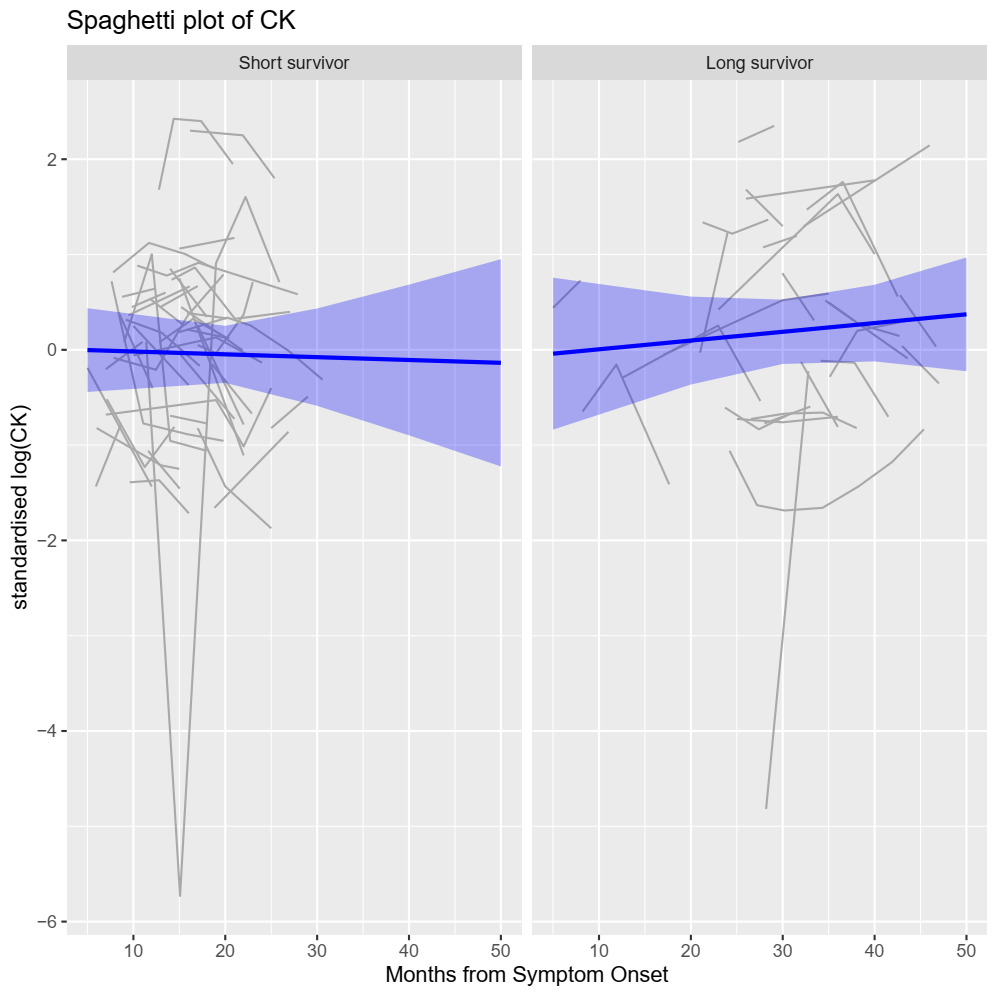


Estimate for fixed effect of Onset site x Time Estimate for fixed effect of Survival length x Time
= -0.014 (95% CI -0.052 ~ 0.025) = 0.012 (95% CI -0.029 ~ 0.053)

## References

1. Williams ER, Bruford A (1970) Creatine phosphokinase in motor neurone disease. Clin Chim Acta 27:53–56. https://doi.org/10.1016/0009-8981(70)90373-6

2. Welch KM, Goldberg DM (1972) Serum creatine phosphokinase in motor neuron disease. Neurology 22:697–701. https://doi.org/10.1212/wnl.22.7.697

3. Edmonds PJ, Ziegler DK (1975) Diagnostic value of serum creatine phosphokinase in motor neuron disease. South Med J 68:1388–1390. https://doi.org/10.1097/00007611-197511000-00016

4. Amrit AN, Anderson MS (1974) Serum creatine phosphokinase in amyotrophic lateral sclerosis. Correlation with sex, duration, and skeletal muscle biopsy. Neurology 24:834–837. https://doi.org/10.1212/wnl.24.9.834

5. Harrington TM, Cohen MD, Bartleson JD, Ginsburg WW (1983) Elevation of creatine kinase in amyotrophic lateral sclerosis. Potential confusion with polymyositis. Arthritis Rheum 26:201–205. https://doi.org/10.1002/art.1780260212

6. Sinaki M, Mulder DW (1986) Amyotrophic lateral sclerosis: relationship between serum creatine kinase level and patient survival. Arch Phys Med Rehabil 67:169–171. https://doi.org/10.1016/0003-9993(86)90064-x

7. Felice KJ, North WA (1998) Creatine kinase values in amyotrophic lateral sclerosis. J Neurol Sci 160 Suppl 1:S30-32. https://doi.org/10.1016/s0022-510x(98)00195-6

8. Lima AF, Evangelista T, de Carvalho M (2003) Increased creatine kinase and spontaneous activity on electromyography, in amyotrophic lateral sclerosis. Electromyogr Clin Neurophysiol 43:189–192

9. Süssmuth SD, Tumani H, Ecker D, Ludolph AC (2003) Amyotrophic lateral sclerosis: disease stage related changes of tau protein and S100 beta in cerebrospinal fluid and creatine kinase in serum. Neurosci Lett 353:57–60. https://doi.org/10.1016/j.neulet.2003.09.018

10. Iłzecka J, Stelmasiak Z (2003) Creatine kinase activity in amyotrophic lateral sclerosis patients. Neurol Sci 24:286–287. https://doi.org/10.1007/s10072-003-0158-3

11. Gibson SB, Kasarskis EJ, Hu N, et al (2015) Relationship of creatine kinase to body composition, disease state, and longevity in ALS. Amyotroph Lateral Scler Frontotemporal Degener 16:473–477. https://doi.org/10.3109/21678421.2015.1062516

12. Chahin N, Sorenson EJ (2009) Serum creatine kinase levels in spinobulbar muscular atrophy and amyotrophic lateral sclerosis. Muscle & Nerve 40:126–129. https://doi.org/10.1002/mus.21310

13. Zhang Y, Huang J-J, Wang Z-Q, et al (2012) Value of muscle enzyme measurement in evaluating different neuromuscular diseases. Clin Chim Acta 413:520–524. https://doi.org/10.1016/j.cca.2011.11.016

14. Chiò A, Calvo A, Bovio G, et al (2014) Amyotrophic Lateral Sclerosis Outcome Measures and the Role of Albumin and Creatinine: A Population-Based Study. JAMA Neurol 71:1134. https://doi.org/10.1001/jamaneurol.2014.1129

15. Rafiq MK, Lee E, Bradburn M, et al (2016) Creatine kinase enzyme level correlates positively with serum creatinine and lean body mass, and is a prognostic factor for survival in amyotrophic lateral sclerosis. Eur J Neurol 23:1071–1078. https://doi.org/10.1111/ene.12995

16. Lu C-H, Allen K, Oei F, et al (2016) Systemic inflammatory response and neuromuscular involvement in amyotrophic lateral sclerosis. Neurol Neuroimmunol Neuroinflamm 3:e244. https://doi.org/10.1212/NXI.0000000000000244

17. Tai H, Cui L, Guan Y, et al (2017) Correlation of Creatine Kinase Levels with Clinical Features and Survival in Amyotrophic Lateral Sclerosis. Front Neurol 8:322. https://doi.org/10.3389/fneur.2017.00322

18. Mirian A, Korngut L (2018) The Utility of the Laboratory Work Up at the Time of Diagnosis of Amyotrophic Lateral Sclerosis. J Neuromuscul Dis 5:35–38. https://doi.org/10.3233/JND-170281

19. Tai H, Cui L, Liu M, et al (2018) Creatine kinase level and its relationship with quantitative electromyographic characteristics in amyotrophic lateral sclerosis. Clin Neurophysiol 129:926–930. https://doi.org/10.1016/j.clinph.2018.01.071

20. Chen X-P, Wei Q-Q, Ou R-W, et al (2021) Creatine kinase in the diagnosis and prognostic prediction of amyotrophic lateral sclerosis: a retrospective case-control study. Neural Regen Res 16:591–595. https://doi.org/10.4103/1673-5374.293159

21. Ito D, Hashizume A, Hijikata Y, et al (2019) Elevated serum creatine kinase in the early stage of sporadic amyotrophic lateral sclerosis. J Neurol 266:2952–2961. https://doi.org/10.1007/s00415-019-09507-6

22. Guo Q-F, Hu W, Xu L-Q, et al (2021) Decreased serum creatinine levels predict short survival in amyotrophic lateral sclerosis. Ann Clin Transl Neurol 8:448–455. https://doi.org/10.1002/acn3.51299

23. Ceccanti M, Pozzilli V, Cambieri C, et al (2020) Creatine Kinase and Progression Rate in Amyotrophic Lateral Sclerosis. Cells 9:E1174. https://doi.org/10.3390/cells9051174
